# Combined Activity of Saponin B Isolated from *Dodonaea viscosa* Seeds with Pesticide Azadirachtin against the Pest *Spodoptera litura*

**DOI:** 10.3390/metabo14010015

**Published:** 2023-12-25

**Authors:** Hang Yu, Boyu Liu, Yuhan Zhao, Jinliang Li, Guoxing Wu, Junhong Ma, Furong Gui, Feng Tao, Xiaojiang Hao, Xiao Ding, Xiaoping Qin

**Affiliations:** 1 State Key Laboratory for Conservation and Utilization of Bioresources in Yunnan, Yunnan Agricultural University, Kunming 650100, China; yuhang@nwafu.edu.cn (H.Y.); 2022610601@stu.ynau.edu.cn (J.L.); chonchon@ynau.edu.cn (G.W.); guifr@ynau.edu.cn (F.G.); 2022610481@stu.ynau.edu.cn (F.T.); 2 State Key Laboratory of Phytochemistry and Plant Resources in West China, Kunming Institute of Botany, Chinese Academy of Sciences, Kunming 650201, China; liuboyu@stu.ynu.edu.cn (B.L.); zhaoyuhan@mail.kib.ac.cn (Y.Z.); haoxj@mail.kib.ac.cn (X.H.); 3 College of Plant Protection, Yunnan Agricultural University, Kunming 650100, China; 4 School of Chemical Science and Technology, Yunnan University, Kunming 650504, China; 5 Yunnan Tobacco Agricultural Science Research Institute, Kunming 650100, China; 2022610596@stu.ynau.edu.cn

**Keywords:** *Dodonaea viscosa*, azadirachtin, contact angle, surface tension, maximum retention, cell membrane permeability

## Abstract

Azadirachtin is regarded as one of the best botanical pesticides due to its broad spectrum of insecticides and low interference with natural enemies. To enhance the effect of azadirachtin and slow down the generation of resistance, the combined activity was studied. Here, we found that *Dodonaea viscosa* saponin B (DVSB) isolated from the seeds of *Dodonaea viscosa* has good combined activity with the azadirachtin. The mixture of DVSB and azadirachtin in a volume ratio of 1:4 had the strongest combined effect against *Spodoptera litura*, with a co-toxicity coefficient (CTC) of 212.87. DVSB exerted its combined activity by affecting the contact angle, surface tension, maximum retention and cell membrane permeability. When mixed with DVSB, the contact angle and surface tension decreased by 30.38% and 23.68%, and the maximum retention increased by 77.15%. DVSB was screened as an effective combined activity botanical compound of azadirachtin upon the control of *S. litura* and highlights the potential application of botanical compounds as pesticide adjuvants in the pest management.

## 1. Introduction

Plant natural products have great significance for integrated pest control. For example, azadirachtin is a tetranortriterpenoid derived from neem (*Azadirachta indica*) that has excellent bioactivity against more than 600 insect species [1,2]. Some flavonoids and sesquiterpene lactones have insecticidal effects through their inhibitory effects on against acetylcholinesterase (AChE) [3,4,5], some sesquiterpenoids and diterpenoid alkaloids have antifeedant effects on lepidoptera pests [6,7,8], and curcumin and zhongshengmycin have good synergistic activity with insecticides [9,10].

With insecticide-resistant pest populations becoming an ever-growing concern, the development of novel synergists is needed. For example, Haitao Chen reports that Beichuang substances and Jiexiaoli substances have synergistic activity with a number of bacteria and that Beichuang substances and Jiexiaoli substances exert synergistic activities by affecting the contact angle and surface tension [11]. Gaofeng Cui reported that curcumin has synergistic activity with avermectin through the enhancement of programmed cell death [9].

*Dodonaea viscosa* (Sapindaceae) is a perennial shrub that grows in tropical and sub-tropical regions. A few studies have been conducted on the chemical constituents of *D. viscosa* and their pharmacological activities. Flavonoids [12], diterpenes, [13,14] and triterpenoids [15] have been isolated from *D. viscosa* and have been proven to have good insecticidal activity. Crude extracts of *D. viscosa* and the active compound *D. viscosa* saponin A (DVSA) showed good antifeedant activity against *S. litura* by influencing medial and lateral sensillum and the γ-aminobutyric acid system [16]. Saponins are a highly diverse group of plant and marine glycosides that are composed of a steroidal or triterpenoidal aglycone (sapogenin) and one or more sugar chains. Many published reports suggest that saponins are a class of compounds with synergistic activity [17,18,19].

*Spodoptera litura* (Fab.) (Noctuidae: Lepidoptera) is one of the most pervasive pests, infesting more than 300 crop species worldwide [20]. Larvae can cause severe damage to soybean, cotton, tobacco, cruciferous vegetables and other economically important crops [21], and feeding-related losses ranging from 26 to 100% are possible in the field [22]. Many field populations of *S. litura* have developed high resistance to multiple insecticides, including organophosphate, carbamate, pyrethroids and several newer chemical insecticides, such as indoxacarb, abamectin, emamectin benzoate, and chlorantraniliprole [23,24,25,26]. Therefore, improved methods of insecticide utilization to manage *S. litura* populations are needed to sustainably suppress this damaging pest.

To our knowledge, the combined activity of pure compounds from *D. viscosa* with azadirachtin has yet to be studied. Based on this context, the primary objective of this study was to identify the major combined active compound of *D. viscosa* and its combined mechanism. This study is expected to aid in the synergy and decreasing of azadirachtin and to better control *S. litura* in the future.

## 2. Materials and Methods

### 2.1. General Experimental Procedures

Azadirachtin (purity, ≥97%) was purchased from Yunnan Zhongke Biological Industry Co., Ltd. (Kunming, China). SF9 cells were purchased from Qingqi Biotechnology Development Co., Ltd. High performance liquid chromatography (HPLC)-grade acetonitrile (J.T. Baker, Phillipsburg, NJ, USA) and ultra-pure water prepared from a Milli-Q purification system (Millipore, Burlington, MA, USA) were used for semipreparative HPLC analysis. Contact Angle and Surface Tension were measured using the JC2000C1 contact angle/surface tension measuring instrument (Shanghai Zhongchen Digital Technology Equipment Co., Ltd., Shanghai, China).

Electrospray ionization (ESI) was recorded on an Agilent 1290 UPLC/6540, and the 1D and 2D NMR spectra were measured on a Bruker 500 MHz spectrometer, with TMS as the internal standard. An RP-18 column (50 μm, YMC Co. Ltd., Kyoto, Japan) gel, MCI gel (75–150 μm, Sci-Bio Chem Co. Ltd., Chengdu, China), and Sephadex LH-20 (40–70 μm, Amersham Pharmacia Biotech AB, Uppsala, Sweden), were used for column chromatography. Semipreparative HPLC was performed on a YMC Luna C18 (5 μm; 10 × 250 mm) reversed-phase column.

### 2.2. Plant Material

Seeds of *D. viscosa* were purchased from Yunnan Ecological Technology Co., Ltd. (Kunming, China). Selected seeds were dried at room temperature and formed into powder using a laboratory mill. A voucher specimen (No. 1906023) was deposited at the State Key Laboratory of Phytochemistry and Plant Resource in West China, Kunming Institute of Botany, Chinese Academy of Sciences (CAS).

### 2.3. Insects

*The S. litura* used in this study were obtained from Yunnan Agricultural University, Kunming, Yunnan, China, and were cultured on cabbage leaves at 25 °C.

### 2.4. Cell Culture

SF9 cell lines were cultured in dulbecco’s modified eagle medium (DMEM) supplemented with 10% fetal bovine serum (Gibco, Thermo Fisher Scientific, Germany), and 1% antibiotics (penicillin 10,000 U/mL, streptomycin 100 mg/mL) (Solarbio, Beijing, China). The cells were maintained at 37 °C in a humidified atmosphere of 5% CO_2_ and were used during their logarithmic growth phase.

### 2.5. Isolation of DVSB

The extract of the seeds of *D. viscosa* (5 kg) was obtained by extraction with ethanol (10 L) three times (3 × 24 h) at room temperature, and the solvent was evaporated in vacuum. The extract solution was suspended in H_2_O (0.5 L) and subjected to gradual extraction with petroleum ether, ethyl acetate, and n-BuOH (3 × 1.5 L). The n-BuOH extract was concentrated at a reduced pressure to obtain the total saponins from *D. viscosa* (TSDV). The TSDV (50.5 g) was chromatographed on an RP-18 column. Elution with water–methanol (97:3–0:100) was performed to yield six fractions (1–6). Fraction 6 (9.6 g) was separated using a Sephadex LH-20 column eluted with CH_3_OH. Six subfractions (6A–6E) were collected. Fraction 6C (6.2 g) was applied to an MCI gel column (CH_3_OH-H_2_O from 1:9 to 8:2) to yield seven fractions (6C1–6C7). Fr. 6C6 (1.2 g) was purified by a semipreparative C18 HPLC column with H_2_O-ACN (4:6) to obtain DVSB (400 mg).

### 2.6. Assays for Nonselective Antifeedant Activity

The nonselective antifeedant activity of *D. viscosa* extract against 4th—instar larvae of *S. litura* was determined using the leaf disc method [27]. A punch was used to make the rapeseed leaf into a leaf disc with a diameter of 1.5 cm. Different concentration gradients of the extract were prepared and used for treatment. Treatment with distilled water served as the control. For each leaf disc, 50 µL of the different concentrations of compound was dripped onto the leaf surface. After 24 h of culture, the leaf area of each tested insect was measured with checkerboard paper. The equation of the antifeedant activity was obtained via linear regression. The AFC_50_ was calculated. AFC_50_ is the concentration when the inhibition rate is 50%. The experiment was conducted three times, and all treatments were performed with ten samples.

### 2.7. Co-Toxicity Coefficient Assays

The Co-toxicity (CTC) of a pesticide mixture was calculated according to Equations (1)–(5):

(1) $$Toxicity\;index\left(TIA\right)=\frac{AFC50\;of\;pesticide\;A}{AFC50\;of\;pesticide\;A}\times 100$$

(2) $$Toxicity\;index\left(TIB\right)=\frac{AFC50\;of\;pesticide\;A}{AFC50\;of\;pesticide\;B}\times 100$$

(3) $$Actual\;toxicity\;index\left(ATI\right)=\frac{AFC50\;of\;pesticide\;A}{AFC50\;of\;pesticide\;mixture}\times 100$$

(4) Theoretical toxicity index (TTI)=TIA×pA+TIB×pB

(5) $$Co-toxicity\left(CTC\right)=\frac{ATI\;of\;pesticide\;mixture}{TTI\;of\;pesticide\;mixture}\times 100$$

where pA and pB represent the mass ratio of pesticide A and pesticide B in the mixture, respectively. A CTC value less than 80 indicates an antagonistic effect, a CTC value between 80 and 120 shows an additive effect, and a CTC value greater than 120 indicates a combined effect.

### 2.8. Contact Angle Assays

DVSB was dropped onto the surface of rape leaves with a pipette, and then the change trend of the contact angle of the drops within 0–40 s was recorded with the video function of the contact angle/surface tension measuring instrument. The contact angle at 20 s was selected as the static contact angle, and the degree of contact angle was calculated using the fitting analysis method. The experiment was conducted three times.

### 2.9. Surface Tension Assays

The surface tension of the agent was measured according to the method described in the national standard [28]. Deionized water was used to prepare DVSB into solutions with different mass concentrations and fully mix them evenly. The micro screw rod on the contact angle/surface tension measuring instrument was used to control the reagent micro feeding, and the micro syringe and the micro screw rod were fixed on the specially designed clamping device to ensure the stability of the hanging drop. The spiral micrometer was adjusted to control the liquid drop, which formed a hanging drop. When the shape of the hanging drop is stable, the hanging drop image data were collected. The experiment was conducted three times.

### 2.10. Maximum Retention Assays

The method for measuring the maximum retention refers to the method of Feng Zhu [29]. A punch was used to make the leaf into a leaf disc with a diameter of 1.5 cm. The leaf disc (m1) was weighed to determine its mass, then immersed completely for approximately 10 s with the mixture of DVSB and azadirachtin. It was then removed until no liquid medicine drops formed, weighed, and the reading was recorded as m2. The experiment was conducted three times. The maximum retention of a pesticide mixture was calculated according to Equation (6):

(6) $$Maximum\;Retention=\frac{m2-m1}{the\;area\;of\;leaf\;disc}$$

### 2.11. Cell Membrane Permeability Assays

Propidium iodide (Coolaber, Beijing, China) was used to detect the cell membrane permeability levels according to the manufacturer’s protocol. In brief, cells were seeded in 6-well plates and cultured with DVSB (5, 10, 15, and 20 µM). After treatment, cells were harvested and washed twice with phosphate buffered saline (PBS) and labeled with 2 µM propidium iodide (PI) at 37 °C for 1 h in the dark. Cells were then collected and the fluorescence intensity of DCF was tested by Bio Tek (Bio Tek, Winooski, VT, USA).

The cytotoxicity caused by DVSB was measured by the 3-(4,5-dimethylthiazol-2-yl) -2,5-diphenyltetrazolium bromide (MTT) assay (Meilunbio, Dalian, China). Cells were seeded in 96-well plates in 100 µL of medium. MTT solution (20 µL) was added to each well and incubated at 37 °C for 4 h. The absorbance at 450 nm was measured on a spectrophotometer.

### 2.12. Statistical Analysis

AFC_50_ values were calculated using GraphPad Prism v7.0.0 and statistical analysis was carried out in SPSS 26. The results presented in the study are given as means ± standard error (SE) from three independent experiments, including 10 repeats for each experiment. The one-way analysis of variance (ANOVA) with comparisons of means using Tukey’s honestly significant difference (HSD) test were used to compare surface tension, contact angle, maximum retention, and cell viability (*p* < 0.05).

## 3. Results

### 3.1. Isolation of Dodonaea viscosa Saponin B

**Figure 1:**
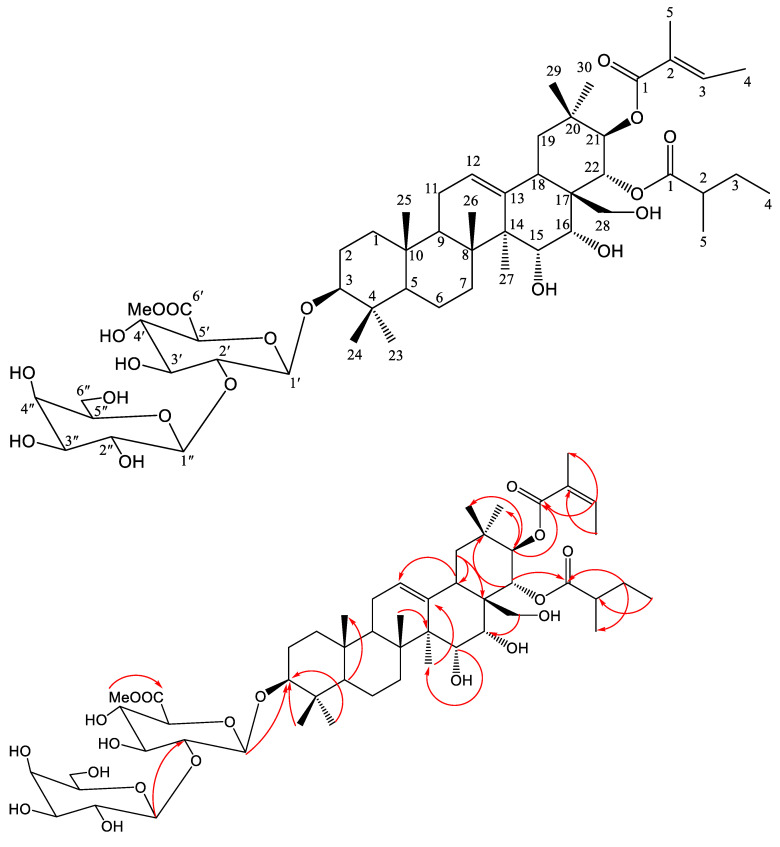
The chemical structure of *D. viscosa* saponin B.

*Dodonaea viscosa* Saponin B (DVSB) was obtained as a white amorphous powder. The positive-ion HR-ESI-MS spectrum of DVSB displayed an [M + Na] + peak at m/z 1057.5497 (calcd. 1047.5499), corresponding to a molecular formula of C_53_H_84_O_19_Na. The IR spectrum showed the presence of carbonyl groups (1715.63 cm^−1^) and hydroxyl groups (3425.39 cm^−1^). The ^13^C NMR (Table 1) spectrum showed the resonances of fifty-three carbons, ascribable to twelve methyl, nine methylene, twenty-one methine, and eleven quaternary carbons, as revealed by the distortionless enhancement by polarization transfer (DEPT) experiment. The ^1^H NMR (Table 1) spectrum showed two olefinic proton signals at δ_H_ 5.50 (Me-12) and δ_H_ 6.06 (Me-C-21-Ang-3) and twelve methyl proton signals at δ_H_ 1.29 (Me-23), 1.11 (Me-24), 0.86 (Me-25), 1.03 (Me-26), 1.84 (Me-27), 1.09 (Me-29), 1.30 (Me-30), 3.70 (C-6-OMe), 2.16 (C-21-Ang-4), 2.03 (C-21-Ang-5), 0.74 (C-22-2MB-4) and 0.86 (C-21-Ang-5). The ^13^C NMR data indicated the presence of the C-COOMe group at δ_C_ 52.5. The other data were comparable to those reported for 60-methylether-*O*-xanifolia-Y_5_ published in the literature, suggesting that DVSB featured the same aglycone present in 60-methylether-O-xanifolia-Y_5_ (Table 1) [30]. The relative configuration of 1 was determined from the HSQC experiment. The HMBC correlations between δ_H_ 6.24 (C-22) and δ_H_ (C-22-2MB-1) suggested that the C-22-2-MB-1 chain attached to C-22; similarly, the C-21-2Ang-1 chain attached to C-21. And the HMBC correlations between δH 3.3 (C-33) and δH 5.0 (C-1′ of GLcA) suggested that the C-33 chain attached to C-1′ of GLcA; in a similar manner, C-1′ of Galactose attached to C-2′ of GLcA. Based on the above analyses, the structure of 1 was established to be 3-*O*-[-D-galactopyranosyl(12)]--D-6′-methyl-glucuronic acid-21β-{(Z)-2-methylbutenoyl}oxy-22α-(2-methylbutanoyl)oxy-3b,15a,16a,21b,22a, 28-hexahydroxylolean-12-ene (Figures S1–S9) and named *Dodonaea viscosa* Saponin B (DVSB), as shown in Figure 1.

### 3.2. Evaluation of the Antifeedant Activities of DVSB and Azadirachtin

To evaluate the antifeedant activity of DVSB and azadirachtin on *S. litura*, the leaf disc method was used. The results related to the feeding area and inhibition rates are shown in Table 2 and Table 3. The feeding area of *S. litura* exposed to the DVSB diet at a concentration of 62.5–1000 μg/mL, was significantly smaller than that exposed to the control diet. These results revealed that DVSB has antifeeding activity. The antifeedant activity regression equation of DVSB was *y* = 0.0006*x* + 0.3025 (R^2^ = 0.9190). The AFC_50_ value of DVSB on fourth—instar larvae was 329.17 μg/mL. The feeding area of *S. litura* exposed to the azadirachtin diet was measured, and the antifeedant activity regression equation of azadirachtin was *y* = 0.000527*x* + 0.4877 (R^2^ = 9702). The AFC_50_ value of azadirachtin on fourth—instar larvae was 23.33 μg/mL.

### 3.3. Evaluation of the Antifeedant Activities of Mixtures DVSB with Azadirachtin

To identify combined activity, the antifeedant bioassay method was performed subsequently. As shown in Table 4, Table 5, Table 6 and Table 7, DVSB and azadirachtin mixed at four ratios showed good antifeedant activity. The antifeedant activity regression equation was y = 0.0059x + 0.2507 (R^2^ = 0.9443) at a volume ratio of 1:4, y = 0.0026x + 0.2644 (R^2^ = 0.9732) at a volume ratio of 2:3, y = 0.0027x + 0.2078 (R^2^ = 0.9016) at a volume ratio of 3:2, and y = 0.0011x + 0.3169 (R^2^ = 0.9643) at a volume ratio of 4:1. The AFC50 of mixtures of four ratios were 42.25 µg/mL, 90.62 µg/mL, 108.22 µg/mL, and 166.46 µg/mL, respectively.

### 3.4. Evaluation of the Antifeedant Activities Co-Toxicity of Mixtures DVSB with Azadirachtin

The co-toxicity coefficient (CTC) is an important indicator for measuring whether pesticides have combined effects. A CTC value less than 80 indicates an antagonistic effect, a CTC value between 80 and 120 shows an additive effect, and a CTC value greater than 120 indicates a combined effect [24]. Since the mixtures of DVSB and azadirachtin have good antifeedant activity, to confirm whether DVSB has a combined effect on azadirachtin, the CTC method was used. The results of the CTC are shown in Table 8. The CTC values of mixture 1, mixture 2, mixture 3 and mixture 4 are greater than 120. These results revealed that DVSB has a combined effect on azadirachtin. The best combined effect for mixtures of DVSB and azadirachtin was observed at a 1:4 volume ratio, with a CTC of 212.87.

### 3.5. Effects of DVSB on Contact Angle

**Figure 2:**
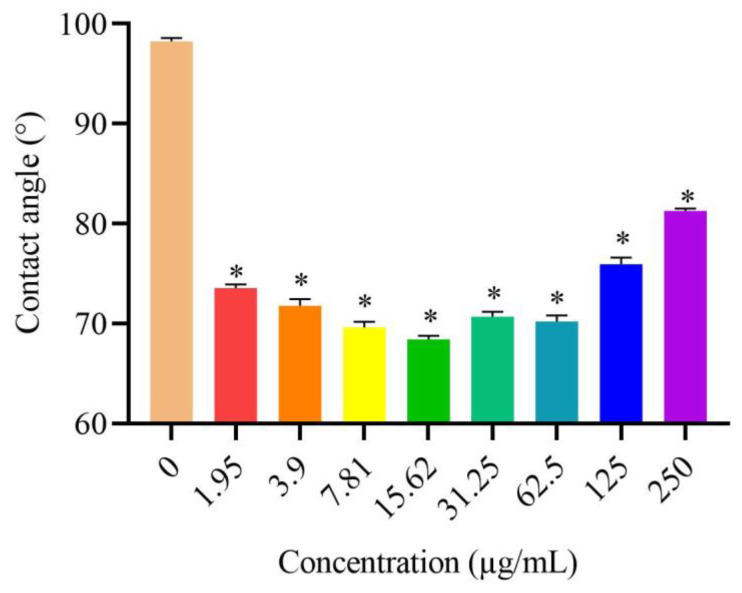
The effects of DVSB on contact angle, * indicates significant differences according to Tukey’s post hoc test, *p* < 0.05.

The contact angle is the included angle between the solid–liquid interface and the gas–liquid interface through the liquid interior at the junction of solid, liquid and gas phases. It is the most direct indicator of the spread ability of pesticide droplets on plant leaves. As shown in Figure 2, the contact angles of the medicine with DVSB were significantly decreased compared to those of the control group. At a concentration of 15.63 µg/mL, the contact angle was decreased by 30.38%. These results suggested that DVSB can decrease the contact angle of the medicine significantly. This is one of the reasons why DVSB has combined activity.

### 3.6. Effects of DVSB on Surface Tension

**Figure 3:**
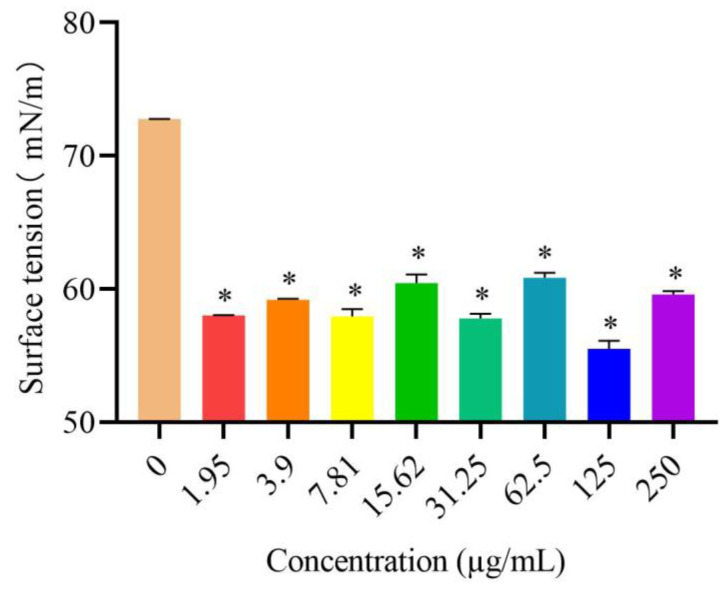
The effects of DVSB on surface tension, * indicates significant differences according to Tukey’s post hoc test, *p* < 0.05.

Surface tension is an important indicator of solid surface energy in solid–liquid interface chemistry which is used to explain liquid’s ability to spread on solid surfaces. The results showed that the surface tension of the medicine added to DVSB was lower than that of the control group. At the concentration of 125 µg/mL, DVSB had the best decrease in surface adsorption in the concentration range of 1.95 µg/mL to 250 µg/mL. The surface tension after adding 125 µg/mL DVSB was decreased by 23.68% compared to that of the control group (Figure 3). These results showed that DVSB can decrease the surface tension of the medicine significantly. This is one of the reasons why DVSB has combined activity.

### 3.7. Effects of DVSB on Maximum Retention

**Figure 4:**
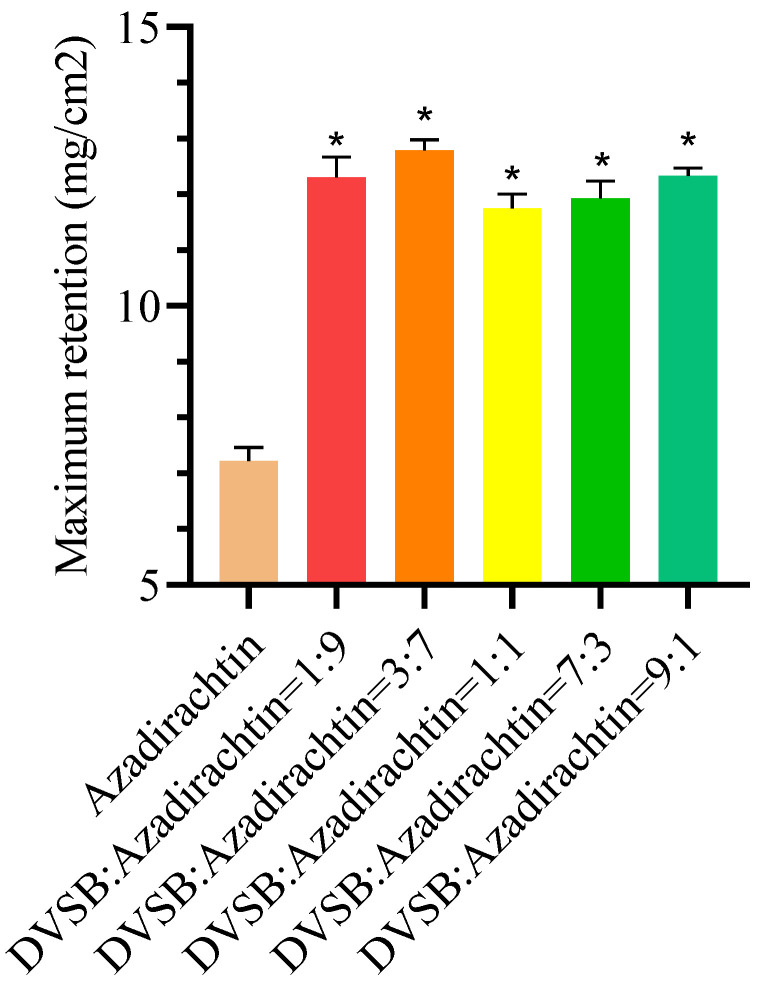
The effects of DVSB on maximum retention. * indicates significant differences according to Tukey’s post hoc test, *p* < 0.05.

The maximum retention (Rm) refers to the maximum content of pesticides that can be adsorbed by the leaves per unit area, which is an important indicator for measuring the quality of pesticides. As shown in Figure 4, the maximum retention of the mixture was higher than that of azadirachtin. When the mixture of DVSB and azadirachtin reached a volume ratio of 3:7, the maximum retention was increased by 77.15%. These results suggest that DVSB can increase the maximum retention of pesticides. This is another reason why DVSB has combined activity.

### 3.8. Effects of DVSB on Cell Membrane Permeability

**Figure 5:**
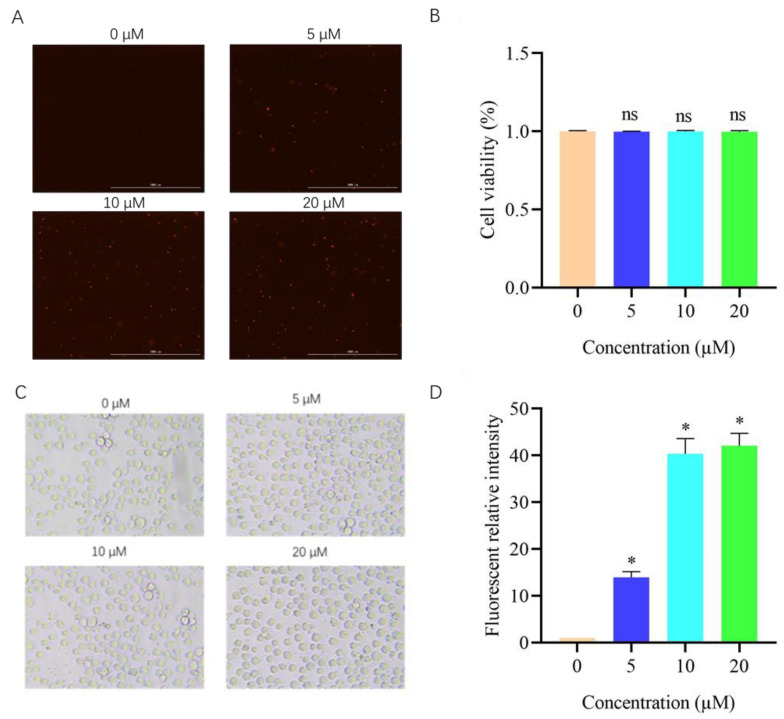
Effect of DVSB on the membrane permeability of SF9 cells. (**A**) shows the effect of DVSB on membrane permeability, (**B**) shows the effect of DVSB on the toxicity of SF9 cells, (**C**) shows the effect of DVSB on the morphology of SF9 cells, (**D**) shows the statistical diagram of the effect of DVSB on membrane permeability, and * indicates significant differences according to Tukey’s post hoc test, *p* < 0.05. ns indicates non-significant differences according to Tukey’s post hoc test, *p* > 0.05.

According to previous studies, saponins can enhance the permeability of the cell membrane [31]. To confirm whether DVSB has a combined effect by increasing the permeability of the cell membrane, the permeability of SF9 cells was measured. PI is a fluorescent probe used to detect the changes in cell membrane permeability when cells are damaged. When cells are living cells, because the cell membrane is complete, PI cannot pass through living cells. When the cell is in apoptosis or necrosis, due to the increased permeability of its cell membrane, propidium iodide (PI) is allowed to enter the cell and can emit red fluorescence when combined with DNA. When the cell is in the normal living cell state, the compound can make PI enter the cell by increasing the permeability of the cell membrane. As shown in Figure 5B,C, the DVSB acted on SF9 cells, and there was no obvious damage to the cells, so it can be considered that the cells are still normal living cells. The MTT results can also showed that the cells were normal living cells. As shown in Figure 5A,D, the red fluorescence of SF9 cells cultured with DVSB was more significant than that of the control group. These results revealed that DVSB can increase the cell membrane permeability, and allow more drugs to enter the cells to have combined activity with azadirachtin.

## 4. Discussion

Saponins are a highly diverse group of plant and marine glycosides that are composed of a steroidal or triterpenoidal aglycone (sapogenin) and one or more sugar chains. Depending on the number of sugar chains attached, they are classified as monodesmosidic (one sugar chain), bisdesmosidic (two sugar chains) and, in rare cases, trisdesmosidic (three sugar chains). Sugar chains mostly comprise of monosaccharides such as glucose, galactose, xylose, rhamnose and glucuronic acid along with numerous others. In previous reports, a number of biological effects have been attributed to saponins, including membrane permeabilizing characteristics [25] and combined activity, and Shan Yin et al. speculate that glycan ligands in the structure of saponins could be pivotal pharmacophores of synergistic activity [32]. In the present study, we isolated the saponin DVSB from *Dodonaea viscosa*, and investigated its combined activity.

Pesticide synergists have become an important method of integrated pest management because of their effectiveness in improving pesticide utilization and reducing environmental pollution. Azadirachtin is a compound with good antifeedant activity against *S. litura*, with the ability to deter feeding in Lepidoptera pests at very low concentrations [33,34]. The result was consistent with our result. Compared to the previous study [35,36], the antifeedant activity of DVSB is not very strong, but we found that DVSB has good combined activity with azadirachtin. The mixture of DVSB and azadirachtin in a volume ratio of 1:4 had the strongest combined effect against *S. litura*, with a co-toxicity coefficient (CTC) of 212.87. The CTC of DVSB and azadirachtin is in the middle level, compared to the previous study [37,38,39].

It is generally believed that the combined mechanisms of synergists include contact angle, surface tension, maximum retention and detoxification enzyme activity. In addition, DVSB cannot decrease detoxification enzyme activity (according to unpublished data from our laboratory), so detoxification enzymes are not the reason for the combined activity of DVSB. In the present study, we found combined activity of DVSB on azadirachtin by affecting the contact angle, surface tension and maximum retention. Haitao Chen et al. found that the combination of imidacloprid with combined agents can lead to improving the spread of imidacloprid droplets on plant leaves [8], which was consistent with our results. Feng Zhu et al. found that surfactant solutions (N-200, N-300, Tween-80, blend of alkyl naphthalene sulfonate and anionic wetting agent, dodecyl trimethyl ammonium bromide (DTAB), and sodium dodecyl sulfate (SDS) can improve pesticide utilization efficiency by affecting surface tension, maximum retention and contact angle [29]. These results were consistent with our results.

In addition, it has been reported that synergists produce combined activity by affecting cell membrane permeability. For example, Jianping Chen et al. found that natural borneol potentiated curcuminoid’s inhibition of HepG2 human hepatocellular carcinoma cells by affecting cell membrane permeability [40]. Zdybicka-Barabas and Agnieszka found that apoLp-III can enhance the permeabilizing activity of lysozyme toward *Escherichia coli* cells and have combined activity with *G. mellonella* lysozyme [41].

In summary, DVSB was screened as an effective combined botanical compound of azadirachtin upon the control of *S. litura*. Surface tension, maximum retention, contact angle and cell membrane permeability-related characteristics were all featured after DVSB was combined with azadirachtin. Thus, surface tension, maximum retention, contact angle, and cell membrane permeability were supposed to be the main combined mechanism of DVSB combined with azadirachtin. This study highlights the potential application of botanical compounds as pesticide adjuvants in pest management.

## 5. Conclusions

In this study, we isolated the combined activity compound and identified it as DVSB. The results show that DVSB has good combined activity with azadirachtin, and the reason for the combined activity is the contact angle, surface tension, maximum retention, and cell membrane permeability.

## Figures and Tables

**Table 1:** NMR spectral data of DVSB.

NO.	δ_H_ *J* (Hz)	δ_C_
1	0.88, m, 1.45, m	39.4
2	2.12, m, 1.87, m	27.0
3	3.3 (dd, 3.9, 11.5)	89.6
4	−	40.0
5	0.79 (d, 12.2)	56.0
6	1.32, m, 1.55 (d, 12.2)	19.2
7	2.12, m, 2.03, m	37.4
8	−	41.9
9	1.70, m	47.6
10	−	37.2
11	1.80, m, 1.89, m	24.5
12	5.50, s	125.8
13	−	144.2
14	−	48.2
15	4.21, m	67.9
16	4.43, m	73.4
17	−	49.0
18	3.07, m	41.4
19	3.07, m, 1.42 (d, 9.6)	47.2
20	−	36.8
21	6.66 (d, 10.2)	78.9
22	6.24 (d, 10.2)	73.6
23	1.29, s	28.5
24	1.11, s	17.2
25	0.86, s	16.3
26	1.03, s	18.0
27	1.84, s	21.7
28	3.76 (d, 9.6) 3.48 (d, 9.6)	63.6
29	1.09, m	29.9
30	1.30, s	20.7
C-3-GlcA		
1′	5.0, s	105.8
2′	4.26, m	84.2
3′	4.32 (t, 9.0)	77.9
4′	4.43, m	73.3
5′	4.06, m	77.5
6′	-	171.0
C-6′-OMe	3.70, s	52.5
GlcA -2-Gal		
1″	5.23 (d, 7.6)	107.7
2″	4.58, m	75.2
3″	4.18, m	75.4
4″	4.70, s	69.9
5″	4.54 (d, 9.7)	77.2
6″	4.41, m, 4.62, m	61.7
C-21-Ang		
1	−	167.9
2	−	129.3
3	6.06, m	139.2
4	2.16 (d, 7.2)	16.6
5	2.03, s	21.6
C-22-2MB		
1	−	176.9
2	2.09, m	42.2
3	1.64, m 1.22, m	27.2
4	0.74 (t, 7.4)	12.3
5	0.86, s	17.5

**Table 2:** Antifeedant activity of DVSB against *Spodoptera litura*.

Concentration (μg/mL)	Feeding Area (mm^2^)	Inhibition Rate (%)
Control check (CK)	1075.14 ± 4.87 ^a^	
62.5	703.36 ± 3.42 ^b^	34.57
125	703.36 ± 3.93 ^b^	34.57
250	663.17 ± 10.72 ^c^	40.18
500	343.83 ± 9.84 ^d^	68.02
1000	160.77 ± 3.93 ^e^	85.05

Feeding area is presented as the mean of ± SE (*n* = 10). Different superscript letters indicate significant differences according to Tukey’s post hoc test, *p* < 0.05.

**Table 3:** Antifeedant activity of azadirachtin against *Spodoptera litura*.

Concentration (μg/mL)	Feeding Area (mm^2^)	Inhibition Rate (%)
Control check (CK)	1075.14 ± 4.87 ^a^	
12.34	559.58 ± 4.64 ^c^	47.95
37.03	556.92 ± 5.81 ^d^	48.2
111	496.26 ± 6.38 ^e^	53.84
333	291.83 ± 4.39 ^f^	72.85
1000	6.02 ± 0.82 ^g^	99.43

The purity of azadirachtin is 97%, and the feeding area is presented as the mean of ± SE (*n* = 10). Different superscript letters indicate significant differences according to Tukey’s post hoc test, *p* < 0.05.

**Table 4:** Antifeedant activity of mixture (DVSB: azadirachtin = 1:4) against *Spodoptera litura*.

Concentration (μg/mL)	Feeding Area (mm^2^)	Inhibition Rate (%)
Control check (CK)	1075.14 ± 4.87 ^a^	
5.44	813.13 ± 20.68 ^b^	24.37
10.87	719.05 ± 10.04 ^c^	33.12
21.74	598.21 ± 12.60 ^d^	44.36
43.5	454.14 ± 7.04 ^e^	57.76
87	266.63 ± 11.23 ^f^	75.2

The mass ratio of mixture is DVSB: azadirachtin = 3.4:1. The purity of azadirachtin is 97%, and the feeding area is presented as the mean of ± SE (*n* = 10). Different superscript letters indicate significant differences according to Tukey’s post hoc test, *p* < 0.05.

**Table 5:** Antifeedant activity of mixture (DVSB: azadirachtin = 2:3) against *Spodoptera litura*.

Concentration (μg/mL)	Feeding Area (mm^2^)	Inhibition Rate (%)
Control check (CK)	1075.14 ± 4.87 ^a^	
9.31	602.88 ± 15.05 ^b^	25.93
18.61	562.69 ± 10.55 ^c^	30.86
37.22	502.40 ± 10.59 ^d^	38.27
74.43	422.02 ± 11.58 ^e^	48.14
148.85	301.44 ± 7.20 ^f^	63

The mass ratio of mixture is DVSB: azadirachtin = 9:1. The purity of azadirachtin is 97%, and the feeding area is presented as the mean of ± SE (*n* = 10). Different superscript letters indicate significant differences according to Tukey’s post hoc test, *p* < 0.05.

**Table 6:** Antifeedant activity of mixture (DVSB: azadirachtin = 3:2) against *Spodoptera litura*.

Concentration (μg/mL)	Feeding Area (mm^2^)	Inhibition Rate (%)
Control check (CK)	1075.14 ± 4.87 ^a^	
13.18	683.26 ± 10.92 ^b^	16.05
26.36	602.88 ± 7.34 ^c^	25.93
52.72	462.21 ± 15.70 ^d^	43.21
105.44	361.73 ± 15.02 ^e^	55.56
210.87	221.06 ± 10.02 ^f^	72.84

The mass ratio of mixture is DVSB: azadirachtin = 20.3:1. The purity of azadirachtin is 97%, and the feeding area is presented as the mean of ± SE (*n* = 10). Different superscript letters indicate significant differences according to Tukey’s post hoc test, *p* < 0.05.

**Table 7:** Antifeedant activity of mixture (DVSB: azadirachtin=4:1) against *Spodoptera litura*.

Concentration (μg/mL)	Feeding Area (mm^2^)	Inhibition Rate (%)
Control check (CK)	1075.14 ± 4.87 ^a^	
17.06	884.22 ± 11.95 ^b^	32.31
34.11	844.03 ± 14.23 ^bc^	35.38
68.23	803.84 ± 10.69 ^c^	38.46
136.47	643.07 ± 12.04 ^d^	50.77
272.93	321.54 ± 11.34 ^e^	60.49

The mass ratio of mixture is DVSB: azadirachtin = 54.3:1. The purity of azadirachtin is 97%, and the feeding area is presented as the mean of ± SE (*n* = 10). Different superscript letters indicate significant differences according to Tukey’s post hoc test, *p* < 0.05.

**Table 8:** Antifeedant activities and CO toxicity coefficient of mixtures of DVSB with azadirachtin.

Mixture (Mass Ratio)	Regression Equation	AFC_50_ (µg/mL)	Correlation Coefficient	Active Toxicity Index	Theoretical Toxicity Index	Co-Toxicity Coefficient
DVSB	*y* = 0.0006*x* + 0.3025	329.17	0.919	100		
Azadirachtin	*y* = 0.000527*x* + 0.4877	23.33	0.9702	1410.93		
DVSB: aza-dirachtin =1:4 (3.4:1)	*y* = 0.0059*x* + 0.2507	38.86	0.9443	847.07	397.93	212.87
DVSB: aza-dirachtin =2:3 (9:1)	*y* = 0.0026*x* + 0.2644	90.62	0.9732	363.24	231.1	157.18
DVSB: aza-dirachtin =3:2 (20.3:1)	*y* = 0.0027*x* + 0.2078	108.22	0.9016	304.17	161.54	188.29
DVSB: aza-dirachtin =4:1 (54.3:1)	*y* = 0.0011*x* + 0.3169	166.46	0.9643	197.75	123.7	159.86

## Data Availability

The data presented in this study are available on request from the corresponding author. The data are not publicly available due to privacy.

## Supplementary Materials

The following supporting information can be downloaded at: https://www.mdpi.com/article/10.3390/metabo14010015/s1, Figure S1: 1H NMR spectrum of *Dodonaea viscosa* Saponin B in pyridine-d5, Figure S2: 13C NMR spectrum of *Dodonaea viscosa* Saponin B in pyridine-d5, Figure S3: HSQC spectrum of *Dodonaea viscosa* Saponin B in pyridine-d5, Figure S4: 1H-1H COSY spectrum of *Dodonaea viscosa* Saponin B in pyridine-d5, Figure S5: HMBC spectrum of *Dodonaea viscosa* Saponin B in pyridine-d5, Figure S6: ROESY spectrum of *Dodonaea viscosa* Saponin B in pyridine-d5, Figure S7: HRESIMS spectrum of *Dodonaea viscosa* Saponin B, Figure S8: IR spectrum of *Dodonaea viscosa* Saponin B, Figure S9: UV spectrum of *Dodonaea viscosa* Saponin B.
