# Supplementary material for: Combined Activity of Saponin B Isolated from Dodonaea viscosa Seeds with Pesticide Azadirachtin against the Pest Spodoptera litura
Source: Metabolites. 2023 Dec 25;14(1):15. doi: 10.3390/metabo14010015 (PMC10820500; doi:10.3390/metabo14010015)
Supplement: Supplementary file 1 [file metabolites-14-00015-s001.zip › metabolites-2719713-supplementary.pdf]

Synergistic Activity of *Dodonaea viscosa* Saponin B Isolated from *Dodonaea viscosa* Seeds

Hang Yu [1,2,3,†], Boyu Liu [2,4,†], Yuhan Zhao [2], Jinliang Li [1,2,3], Guoxing Wu [1,3], Junhong Ma [5], Furong Gui [1,3], Feng Tao [1,3], Xiaojiang Hao [2], Xiao Ding [2,*], and Xiaoping Qin [1,3*]

[1] *State Key Laboratory for Conservation and Utilization of Bioresources in Yunnan, Yunnan Agricultural University, Kunming 650100, China*

[2] *State Key Laboratory of Phytochemistry and Plant Resources in West China, Kunming Institute of Botany, Chinese Academy of Sciences, Kunming 650201, China*

[3] *College of Plant Protection, Yunnan Agricultural University, Kunming 650100, China*

[4] *School of Chemical Science and Technology, Yunnan University, Kunming 650504, China*

[5] *Yunnan Tobacco Agricultural Science Research Institute, Kunming 650100, China*

[†] These authors have contributed equally to this work.

[*] Correspondence: dingxiao@mail.kib.ac.cn (X.D.); 2005019@ynau.edu.cn (X.Q.)

Contents of Supporting Information

Figure S1 ^{1}H NMR spectrum of *Dodonaea viscosa* Saponin B in pyridine-d_5

Figure S2 ^{13}C NMR spectrum of *Dodonaea viscosa* Saponin B in pyridine-d_5

Figure S3 HSQC spectrum of *Dodonaea viscosa* Saponin B in pyridine-d_5

Figure S4 ^{1}H-^{1}H COSY spectrum of *Dodonaea viscosa* Saponin B in pyridine-d_5

Figure S5 HMBC spectrum of *Dodonaea viscosa* Saponin B in pyridine-d_5

Figure S6 ROESY spectrum of *Dodonaea viscosa* Saponin B in pyridine-d_5

Figure S7 HRESIMS spectrum of *Dodonaea viscosa* Saponin B

Figure S8 IR spectrum of *Dodonaea viscosa* Saponin B

Figure S9 UV spectrum of *Dodonaea viscosa* Saponin B

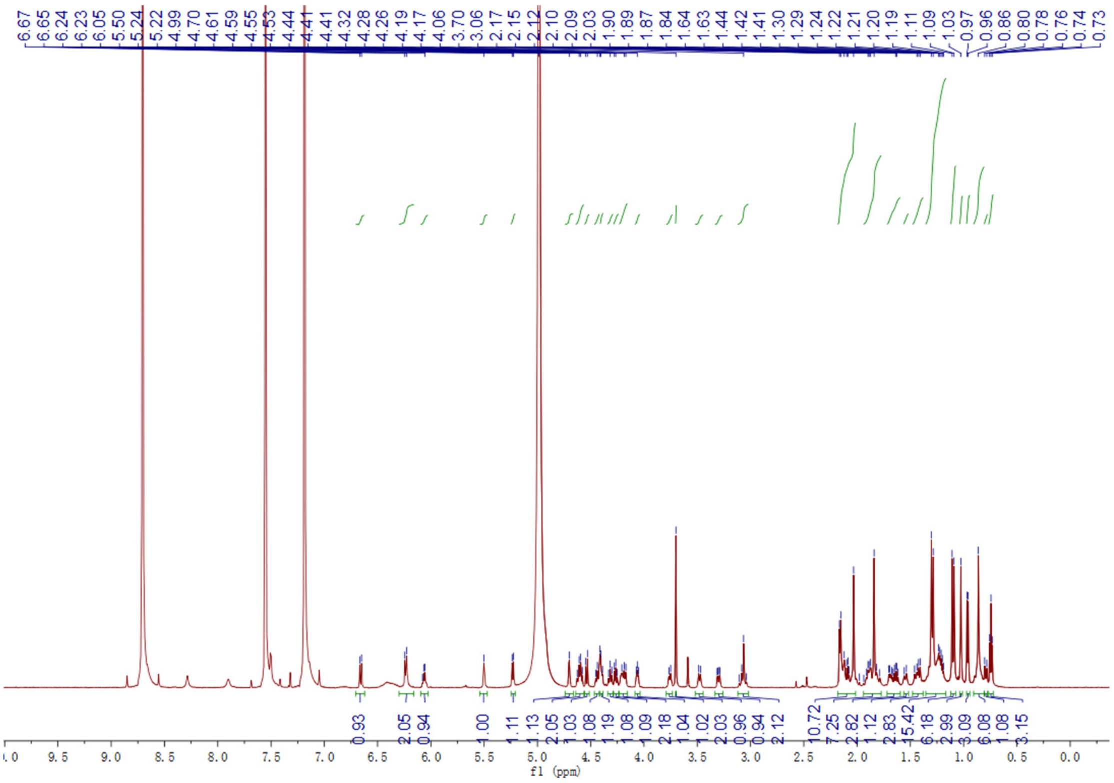

Figure S1 ^{1}H NMR spectrum of *Dodonaea viscosa* Saponin B in pyridine-*d5*

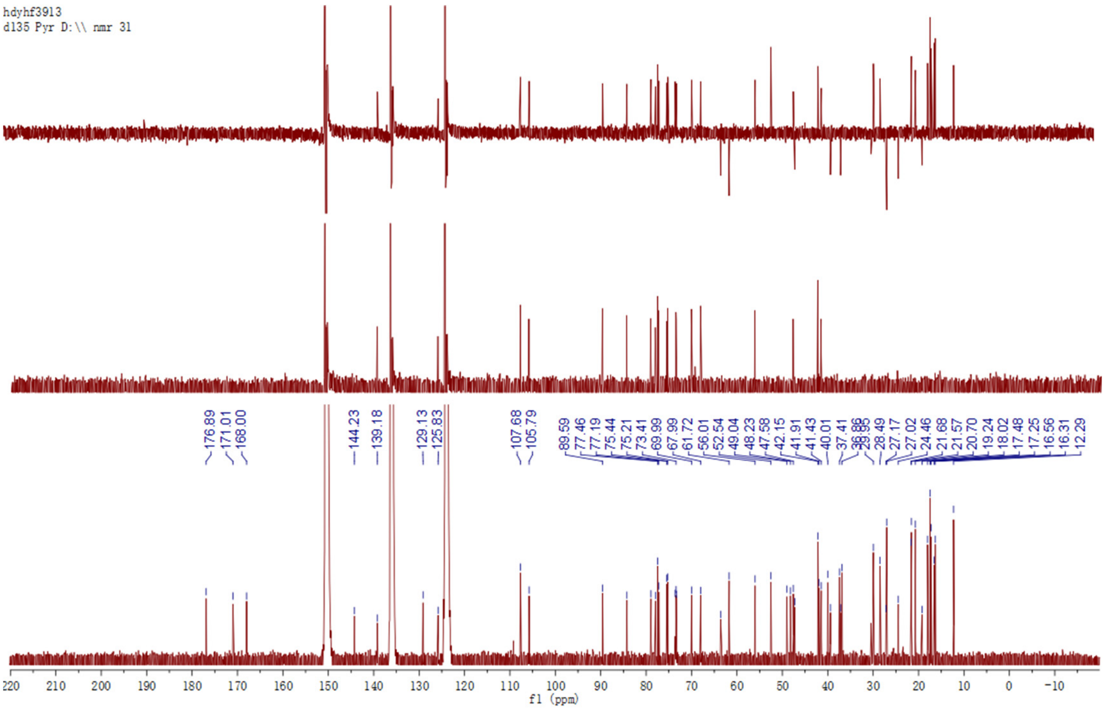

Figure S2 ^{13}C NMR spectrum of *Dodonaea viscosa* Saponin B in pyridine-*d5*

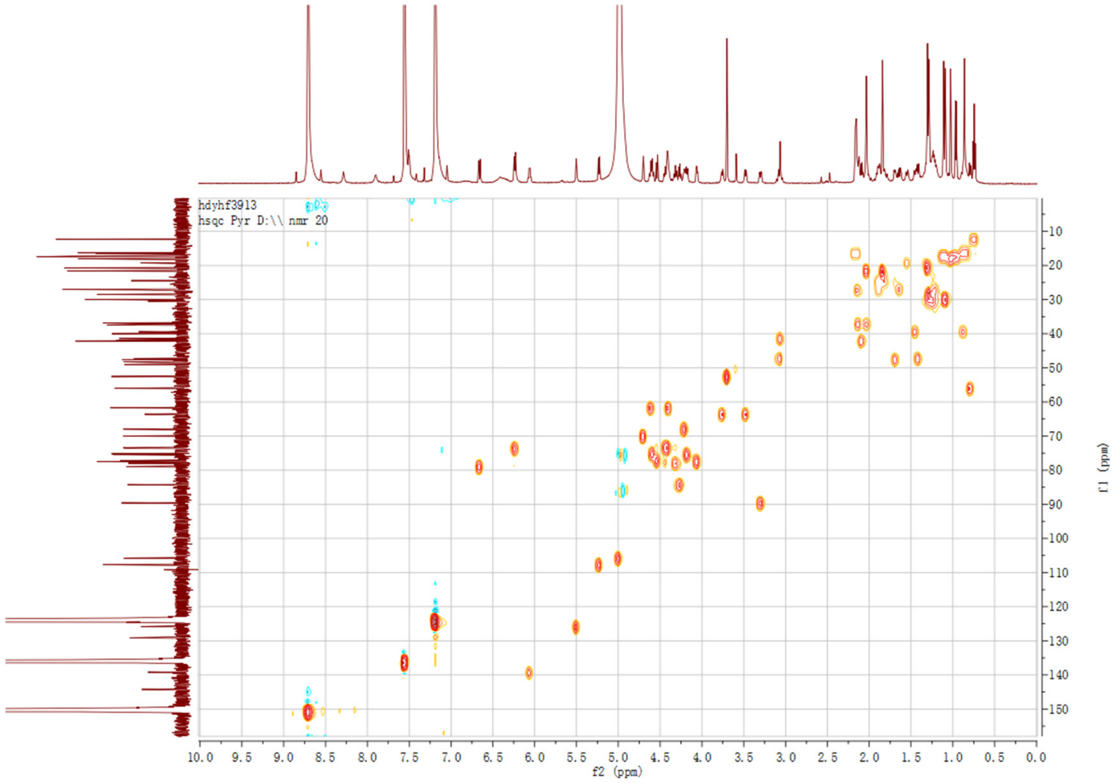

Figure S3 HSQC spectrum of *Dodonaea viscosa* Saponin B in pyridine-*d*5

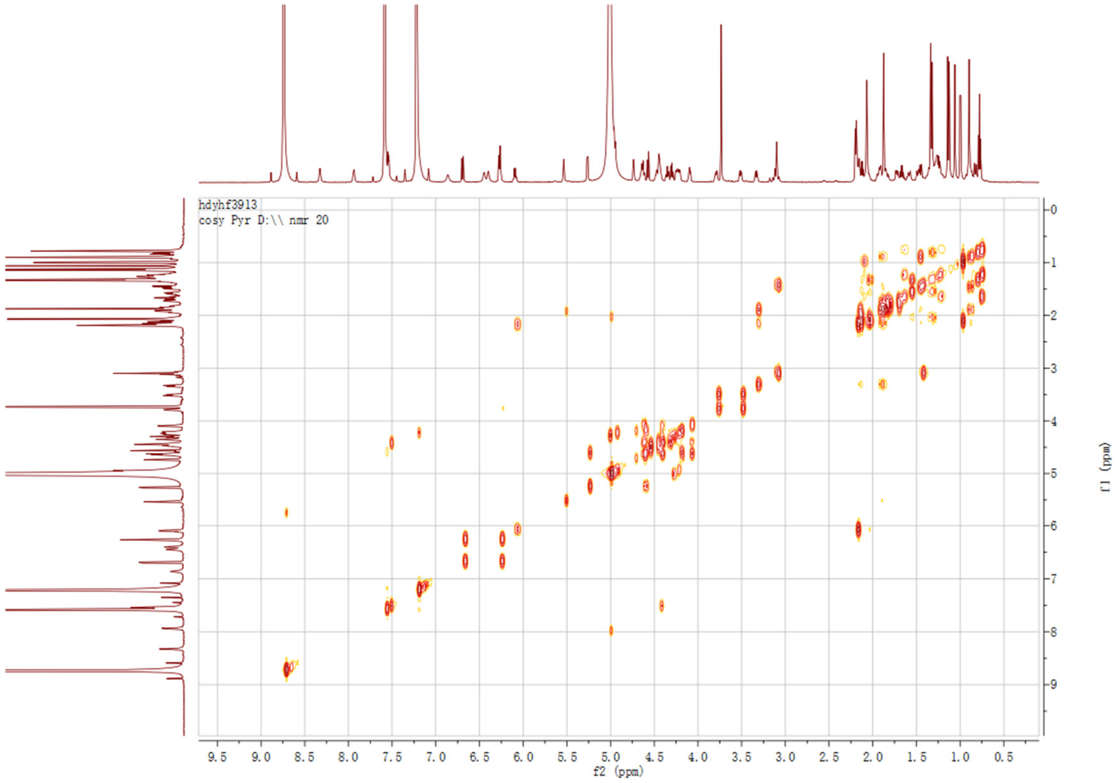

Figure S4 ^{1}H-^{1}H COSY spectrum of *Dodonaea viscosa* Saponin B in pyridine-d_5

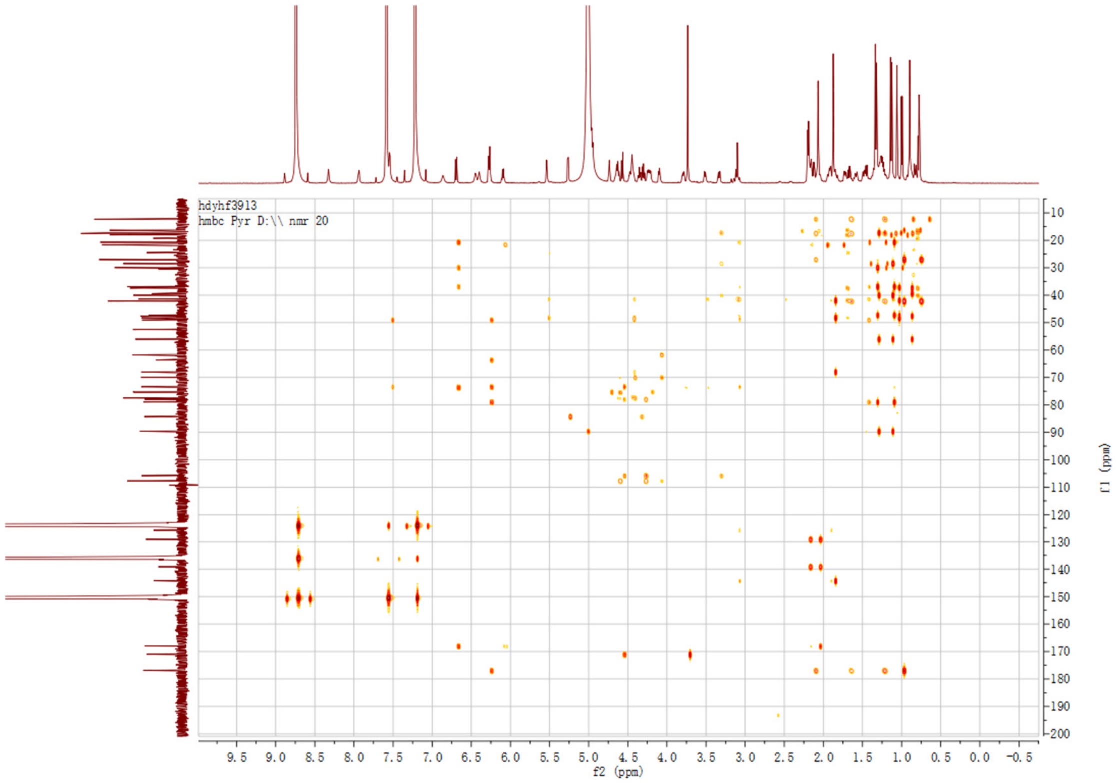

Figure S5 HMBC spectrum of *Dodonaea viscosa* Saponin B in pyridine-d_5

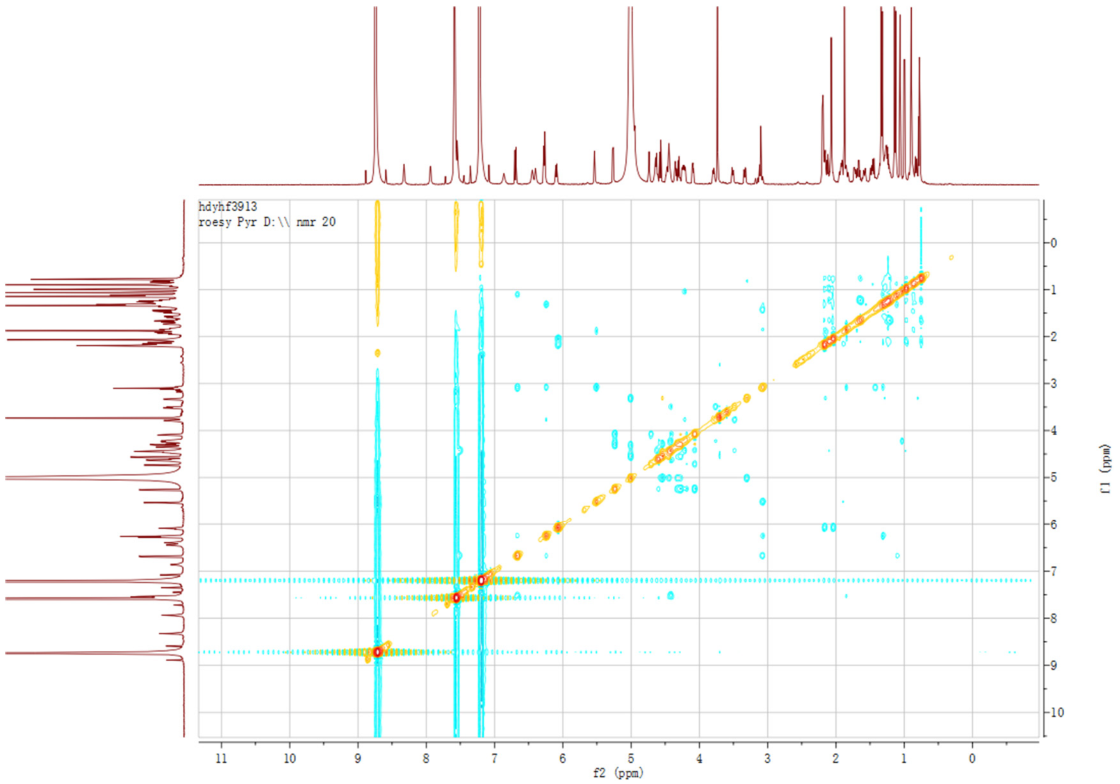

Figure S6 ROESY spectrum of *Dodonaea viscosa* Saponin B in pyridine-d_5

Qualitative Analysis Report

**Data Filename**	hdyhf39-1-3.d	**Sample Name**	hdyhf39-1-3
**Sample Type**	Sample	**Position**	P1-A3
**Instrument Name**	Instrument 1	**User Name**	
**Acq Method**	s.m	**Acquired Time**	3/30/2021 2:15:30 PM
**IRM Calibration Status**	Success	**DA Method**	PCDL.m
**Comment**			

Sample Group **Info.**
Acquisition SW 6200 series TOF/6500 series
Version Q-TOF B.05.01 (B5125.2)

User Spectra

Fragmentor Voltage	Collision Energy	Ionization Mode
135	0	ESI

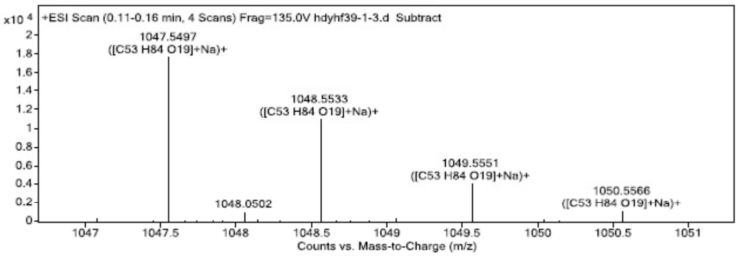

Peak List

m/z	z	Abund	Formula	Ion
111.1811	1	19332.08		
112.1873	1	87122.13		
274.274	1	23268.51		
318.3004	1	16833.15		
526.3909	1	11956.44		
1047.5497	1	17682.78	C53 H84 O19	(M+Na)+
1109.6422	1	171479.31		
1110.6451	1	106401.05		
1111.648	1	39008.38		
1112.6511	1	11342.44		

Formula Calculator Element Limits

Element	Min	Max
C	3	60
H	0	150
O	0	30

Formula Calculator Results

Formula	CalculatedMass	CalculatedMz	Mz	Diff. (mDa)	Diff. (ppm)	DBE
C53 H84 O19	1024.5607	1047.5499	1047.5497	0.20	0.19	12.0000

--- End Of Report ---

Figure S7 HRESIMS spectrum of *Dodonaea viscosa* Saponin B

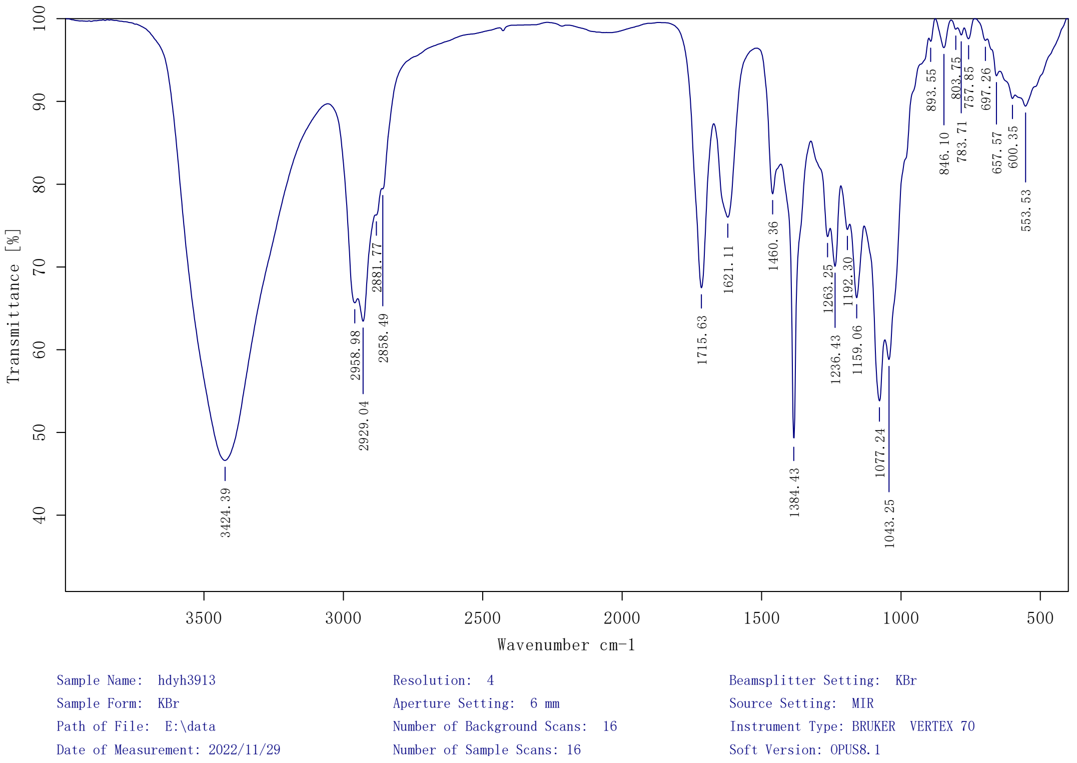

Figure S8 IR spectrum of *Dodonaea viscosa* Saponin B

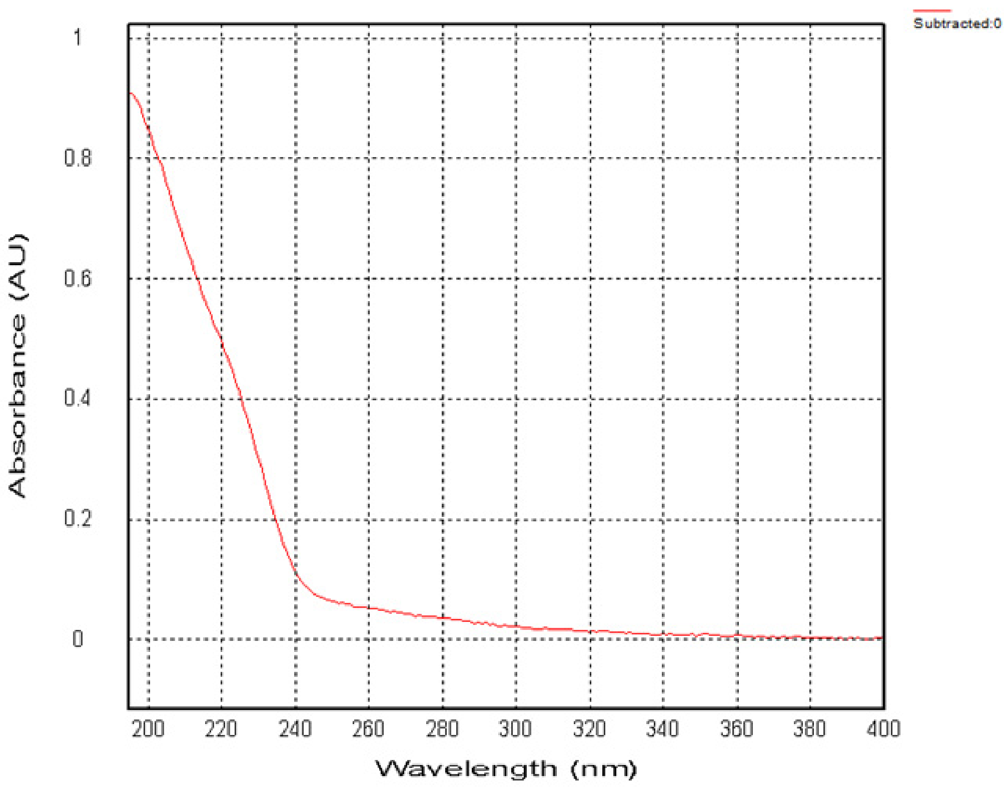

Figure S9 UV spectrum of *Dodonaea viscosa* Saponin B
